# Association of polygenic risk for schizophrenia with fast sleep spindle density depends on pro-cognitive variants

**DOI:** 10.1007/s00406-022-01435-3

**Published:** 2022-06-20

**Authors:** Claudia Schilling, Lea Zillich, Michael Schredl, Josef Frank, Emanuel Schwarz, Michael Deuschle, Andreas Meyer-Lindenberg, Marcella Rietschel, Stephanie H. Witt, Fabian Streit

**Affiliations:** 1 Central Institute of Mental Health, Sleep Laboratory, Medical Faculty Mannheim, Heidelberg University, J5, 68159 Mannheim, Germany; 2 Central Institute of Mental Health, Department of Genetic Epidemiology in Psychiatry, Medical Faculty Mannheim, Heidelberg University, Mannheim, Germany; 3 Central Institute of Mental Health, Department of Psychiatry and Psychotherapy, Medical Faculty Mannheim, Heidelberg University, Mannheim, Germany; 4 Central Institute of Mental Health, Center for Innovative Psychiatry and Psychotherapy, Medical Faculty Mannheim, Heidelberg University, Mannheim, Germany

**Keywords:** Sleep spindles, Genetics, Schizophrenia, Polygenic score, Cognition

## Abstract

Cognitive impairment is a common feature in schizophrenia and the strongest prognostic factor for long-term outcome. Identifying a trait associated with the genetic background for cognitive outcome in schizophrenia may aid in a deeper understanding of clinical disease subtypes. Fast sleep spindles may represent such a biomarker as they are strongly genetically determined, associated with cognitive functioning and impaired in schizophrenia and unaffected relatives. We measured fast sleep spindle density in 150 healthy adults and investigated its association with a genome-wide polygenic score for schizophrenia (SCZ-PGS). The association between SCZ-PGS and fast spindle density was further characterized by stratifying it to the genetic background of intelligence. SCZ-PGS was positively associated with fast spindle density. This association mainly depended on pro-cognitive genetic variants. Our results strengthen the evidence for a genetic background of spindle abnormalities in schizophrenia. Spindle density might represent an easily accessible marker for a favourable cognitive outcome which should be further investigated in clinical samples.

**Supplementary Information:**

The online version contains supplementary material available at 10.1007/s00406-022-01435-3.

## Introduction

Schizophrenia is one of the most debilitating psychiatric disorders and has a strong genetic background [1]. It is a highly polygenic disorder, i.e., a large number of common variants contribute each with a small effect to the disorder risk [1]. Recent genome-wide association studies (GWAS) for schizophrenia identified genetic variation in more than 100 loci being associated with schizophrenia [2–4].

Cognitive impairment is a common feature in schizophrenia and has been reported as the strongest prognostic factor for long-term outcome [5]. GWAS reveal extensive genetic overlap between schizophrenia and intelligence [6, 7] with the direction of the majority of overlapping loci representing a higher risk for schizophrenia and poorer cognitive performance [6]. Schizophrenia is a highly heterogeneous disorder with variation in cognitive functioning that is partly explained by genetic factors [8, 9]. Recently, genetic variance associated with educational attainment—often used in large studies as a proxy for intelligence/cognition—has been shown to aid in dissecting genetic heterogeneity of schizophrenia [10]. The authors found strong genetic overlap between educational attainment and schizophrenia, with associated variants showing both concordant and discordant effects, and the results indicating at least two disease subtypes of schizophrenia, one related to high educational attainment and resembling bipolar disorder, and the other closer to a cognitive disorder, independent of bipolar disorder [10]. In an independent sample of patients with schizophrenia, genetic risk variants for schizophrenia concordant for educational attainment showed significant associations with global assessment of functioning and negative symptoms not detected using all schizophrenia risk variants or the ones discordant with educational attainment [10]. Thus, identifying a subset of variants associated with schizophrenia and a concordant effect on cognitive function may aid in characterizing clinically differing disease subtypes. In this perspective, the fast sleep spindle phenotype may be helpful for differentiating between these subtypes, especially taking into account its strong genetic background [11–13], its association with genetic risk for schizophrenia [14–16] and ample evidence for an association with intellectual capacities [17–22].

Sleep spindles are brief bursts of oscillatory EEG activity within the sigma frequency range of NREM sleep [23]. They are a defining feature of stage 2 sleep [24] and also occur in slow wave sleep. Studies investigating the heritability of sleep spindles indicate a substantial genetic background [11–13]. A study characterizing sleep spindles in 415 related subjects estimated spindle density to have a heritability of ~ 0.45 [12]. Also, the heritability of the NREM sleep EEG frequency band between 8 and 15.75 Hz containing sleep spindles has been estimated to be up to 0.96 in a twin study [11]. Two types of sleep spindles can be distinguished depending on their frequency (9–12 Hz slow spindles, 12–15 Hz fast spindles) and their recruitment of partially segregated cortical networks [23, 25, 26]. It has been shown that spindles play an important role in cognitive processes as they facilitate synaptic plasticity [27] and correlate with intellectual capacities [17–22] and with memory consolidation in healthy subjects [19, 28–31], patients with schizophrenia [32–34], and healthy first-degree relatives (FDR) of patients with schizophrenia [15]. Recently, genetic variation at *ZNF804A*, which is robustly associated with schizophrenia, has been correlated with altered cognitive task-dependent dynamics of sleep oscillations including spindles [35]. The neuroanatomical substrate of spindle generation is the thalamo-cortical network [36], which is implicated in functional abnormalities in schizophrenia [37–39]. In patients with chronic and with first-episode schizophrenia as well as in healthy FDR fast sleep spindle density has repeatedly been shown to be reduced [15, 16, 32, 33, 40–46]. Most data on the sleep spindle deficit in schizophrenia refer to fast spindles. In a study investigating both spindle types in schizophrenia and FDR slow spindles were spared from the spindle deficit [15].

Polygenic score (PGS) analyses represent a powerful tool to investigate the association of the genetic predisposition for a disorder or trait with other phenotypes of interest. PGS estimation uses GWAS results to predict genetic risk for each individual in an independent genotyped sample, by multiplying the allele count of each genotyped variant with its effect size in the respective GWAS [47]. Recently, Merikanto and colleagues investigated whether the polygenic risk score for schizophrenia (SCZ-PGS) based on the GWAS by Ripke et al. [2] was associated with sleep spindle activity in a sample of 150 healthy adolescents [14].

Their findings showed fast spindle density and amplitude in the central derivation to be positively correlated with schizophrenia-related genetic risk, thus in the opposite direction to all previous studies in chronic schizophrenia patients, unmedicated first-episode patients and FDR and to recent data from an animal model for schizophrenia [48]. This raises the question of how to explain the apparently contradictory findings with respect to the direction of the association between spindle activity and genetic risk for schizophrenia in healthy [14] versus clinical samples [15, 16, 32, 33, 40–46]. In view of the significant genetic overlap between schizophrenia and cognition [6, 7] and the strong association between spindle activity and cognitive function [17–22], stratification of the genetic risk for schizophrenia into concordant and discordant cognitive genetic variants may help to elucidate the nature of the association of the spindle phenotype with the genetic background of schizophrenia.

In the present study, we aimed (I) to analyze the association of the fast spindle density phenotype with the polygenic risk for schizophrenia in a sample of 150 healthy subjects and (II) to assess the role of the genetic basis of intelligence for this association by separately investigating the subset of single-nucleotide polymorphisms (SNPs) with concordant effects for schizophrenia and intelligence [49] and the subset of discordant SNPs. As the Merikanto study [14] reported an association of slow spindle activity with genetic variability in *CACNA1I*, a schizophrenia risk gene known to be implicated in spindle generation [50–52], we exploratively additionally aimed to replicate this finding.

## Methods

### Procedures

Participants had two nights of polysomnography in the sleep laboratory of the Central Institute of Mental Health (CIMH), Mannheim, Germany. Data of the second night were used for analysis, whereas the first night served as an adaptation night and for the exclusion of previously undiagnosed sleep disorders.

### Study participants

Inclusion criteria were age 18–60 years, good subjective sleep quality, absence of any pre-diagnosed sleep disorder or neurological or psychiatric disorder. Exclusion criteria comprised sleep curtailment prior to the study, severe health problems, history of substance abuse, current medication intake and circadian abnormalities including shift work, recent travels across time zones, or advanced or delayed sleep–wake rhythms. Participants received monetary compensation for participation in the study.

The study sample comprised 150 healthy subjects of European ancestry (93 women; age 30.9 ± 11.6 years).

### Polysomnographic sleep analysis

Polysomnography was performed using a Schwarzer Comlab 32 polysomnograph (Schwarzer GmbH, Munich, Germany) with standard montage according to the criteria of the American Academy of Sleep Medicine [24]. In addition to electroencephalography (EEG) in six derivations (F4–A1, C4–A1, O2–A1, F3–A2, C3–A2 and O1–A2) this included bilateral electrooculography, chin electromyography, surface electromyography of bilateral tibialis anterior muscles, ECG recording and recording of respiratory variables. EEG sampling rate was 256 Hz. Sleep stage scoring and detection of arousals were performed visually according to the criteria of the American Academy of Sleep Medicine [24]. Polysomnographic sleep characteristics are given in Table 1.

**Table 1:** Sleep characteristics and spindle parameters (means ± SD)

	Healthy subjects (n = 150)
Polysomnographic sleep parameters	
Sleep latency (min)	19.5 ± 15.7
TST (min)	393.1 ± 41.2
SPT (min)	426.9 ± 33.4
Sleep efficiency (%)	87.2 ± 7.9
WASO (min)	33.8 ± 29.5
Stage 1 sleep (min)	40.5 ± 22.6
Stage 1 sleep (%)	9.5 ± 5.3
Stage 2 sleep (min)	223.6 ± 42.4
Stage 2 sleep (%)	52.4 ± 9.3
SWS (min)	60.4 ± 41.6
SWS (%)	14.3 ± 10.0
REM sleep (min)	68.3 ± 23.5
REM sleep (%)	15.9 ± 5.2
REM sleep latency (min)	96.0 ± 55.6
REM density	17.9 ± 7.5
Arousal index (n/h TST)	7.1 ± 4.8
PLM index (n/h SPT)	2.4 ± 4.3
Spindle parameters	
Fast spindle characteristics	
Density (number/30 s)	2.22 ± 0.38
Duration (s)	0.82 ± 0.05
Amplitude (µv)	27.56 ± 7.14
Peak frequency (Hz)	13.46 ± 0.57
Slow spindle characteristics (*n* = 140)^a^	
Density (number/30 s)	1.50 ± 0.39
Duration (s)	0.80 ± 0.05
Amplitude (µv)	28.26 ± 7.32
Peak frequency (Hz)	10.84 ± 0.79

*TST* total sleep time, *SPT* sleep period time, *WASO* wake after sleep onset, *SWS* slow wave sleep, *REM* rapid eye movement, *PLM* periodic limb movements

^a^Due to absence of detectable slow spindle peak in ten individuals

### Sleep spindle detection

Fast and slow sleep spindles were analyzed during NREM sleep stages N2 and N3 using the C3-A2 derivation. The 30-s sleep epochs had to be free of artifacts and arousals in order to be included. Artifacts and arousals were identified by visual inspection. Discrete spindle events were automatically detected using a custom-made software tool (SpindleToolbox, version 3) using MATLAB R2009b (The MathWorks, Natick, Massachusetts) based on an algorithm adopted from previous studies [53, 54] and described in Schilling et al. [55]. In brief, power spectra of each participant were calculated, enabling the user to visually detect each individual’s spindle peak for fast and slow spindles. The signal was then band-pass filtered in the range ± 1.5 Hz around the detected spindle peak, and the root mean square (RMS) was calculated for each 200-ms interval of the filtered signal. A spindle was detected if the RMS signal exceeded a threshold of 1.5 standard deviations of the filtered signal for the duration of 0.5–3 s. Cases in which no slow spindle peak was visually detectable in the power spectrum were excluded from slow spindle analysis (10 cases), resulting in a sample of 140 subjects for slow spindle analysis. Fast spindle peaks were detectable in all 150 subjects. Spindle density was defined as the number of sleep spindles detected per sleep epoch of 30 s of all NREM 2 and 3 sleep epochs free of artifacts and was used as the primary quantitative measure of spindle activity. Morphological characteristics of individual spindle events (spindle amplitude and spindle duration) were additionally analyzed in an exploratory way: Spindle duration was defined as the interval between the threshold crossing points of each spindle. Spindle amplitude was calculated as the maximal spindle voltage after band-pass filtering. Spindle parameters are given in Table1.

### Genotyping and quality control

DNA was extracted from whole blood. Genome-wide genotyping was performed using Global Screening Array 1.0 (Illumina, Inc., San Diego, CA, USA) at the Life&Brain facilities, Bonn, Germany. Quality control and filtering was performed using PLINK 1.9 [56] and R statistical environment, version 3.5.1, removing participants with > 0.02 missingness, heterozygosity rate >|.2|, and sex-mismatch. SNPs with a minor allele frequency of < 0.01, deviating from Hardy–Weinberg equilibrium (HWE) with a *p* value of < 10^–6^ and missing data > 0.02 were removed. Relatedness and population structure were filtered based on a SNP-set filtered for high quality (HWE *p* > 0.02, MAF > 0.20, missingness = 0), and LD pruning (pairwise *r*^2^ < 0.1 within a 200 SNP window). If subjects were cryptically related ($\hat{\pi }$ > 0.20), one subject was excluded at random. Control for population stratification was performed by generating principal components and outliers, defined as deviating more than 6 SD on one of the first 20 principal components were excluded. Quality control resulted in a data set of 140 individuals and 481,956 SNPs for genetic analyses on fast spindle activity. In eight out of these 140 individuals, no slow spindle peak was detectable, thus resulting in 132 individuals for genetic analyses on slow spindle activity.

### Polygenic scores

SCZ-PGS were calculated using PRSice 2.1.6 [57] based on GWAS data from the Psychiatric Genomics Consortium (Cases: *n* = 36,989, Controls: *n* = 113,075; [2]). The summary statistics were filtered, excluding SNPs with an info score < 0.9. PGS were calculated for the default *p* value thresholds (PT; 0.001, 0.05, 0.1, 0.2, 0.3, 0.4, 0.5, 1). The PT corresponds to the minimum p-value threshold in the discovery GWAS required for inclusion of the SNPs to be included in the calculation of the PRS, e.g., PT of 1 considers all SNPs for the calculation, PT of 0.001 considers all SNPs with a *p* value smaller than 0.001 in the original GWAS. Selecting PGS at different thresholds, represent a trade-off, with PGS at more liberal PTs having a higher probability to include all relevant SNPs, but also an increased amount of random noise.

To explore the contribution of the genetic basis of intelligence to fast spindle density and its association with SCZ-PGS, we first calculated PGS for a GWAS of intelligence [49] and tested its association with fast spindle density. In a second step and in orientation to analyses presented in Bansal et al. [10], we split the SNPs of the SCZ GWAS into SNPs that either had concordant effects on SCZ and IQ (i.e., SNPs associated with increased Risk for SCZ (OR > 1) and higher IQ (beta > 0)/lower risk for SCZ (OR < 1) and lower IQ (beta < 0); *n*_SNPs_ = 2,823,196), and SNPs with discordant effects (i.e., SNPs associated with increased risk for SCZ (OR > 1) and lower IQ (beta < 0)/lower risk for SCZ (OR < 1) and higher IQ (beta > 0); *n*_SNPs_ = 3,067,373). SCZ-IQ-concordant-PGS and SCZ-IQ-discordant-PGS were then calculated based on those filtered SNP-sets as described above using the *p* values and effect sizes reported for each of the included SNPs in the SCZ GWAS.

To test for the specificity of the association of SCZ with fast spindle density, we additionally calculated PGS for bipolar disorder (Cases: *n* = 20,352, Controls: *n* = 31,358) [58], and PGS for depression (Cases: *n* = 170,756, Controls: *n* = 329,443; excluding 23andMe) [59].

### Explorative additional analysis of *CACNA1I* gene association with spindle density

In analogy to the Merikanto study [14], we exploratively generated a SCZ-PGS limited to variants in the *CACNA1I* gene including 20 kilo bases (kb) down and upstream (Chr 22; bp 39,946,758–40,105,740). To complement the results of the *CACNA1I*-based SCZ-score, we performed a gene-based association test for *CACNA1I* using MAGMA v1.08 [60] to assess whether fast and slow spindle density was associated with genetic variation in *CACNA1I* at a gene level independent of their implication in SCZ, controlling for sex, age and the first ten principle components, using the same ± 20 kb borders.

### Statistical analysis

The prediction accuracy of all PGS was assessed in PRSice 2.1.6 using the *R*^2^ statistics attributable to the PGS, which was computed as the increase in the coefficient of determination (*R*^2^) when the PGS was added to the linear model predicting the respective sleep spindle characteristics including sex, age and the first ten principle components of population stratification as covariates [57].

All reported tests were two tailed with an *α* level of *p* < 0.05.

## Results

### Association of spindle activity with genome-wide polygenic score for schizophrenia

**Fig. 1:**
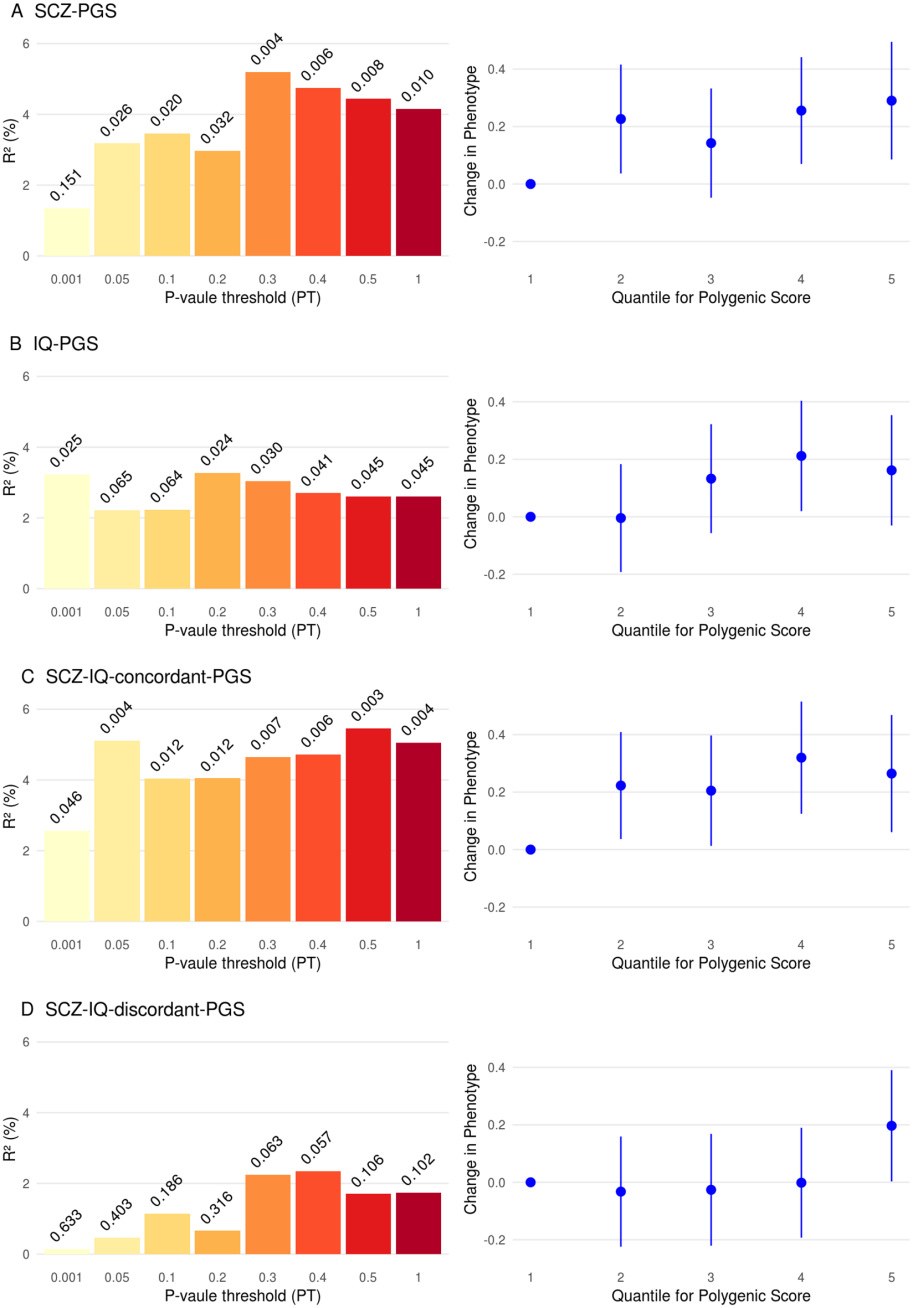
Association of fast spindle density with polygenic scores. **A** Fast spindle density predicted by schizophrenia polygenic score (SCZ-PGS). **B** Fast spindle density predicted by intelligence polygenic score (IQ-PGS). **C** Fast spindle density predicted by polygenic score based on variants with concordant effects on SCZ and intelligence (SCZ-IQ-concordant-PGS). **D** Fast spindle density predicted by polygenic score based on variants with discordant effects on SCZ and intelligence (SCZ-IQ-discordant-PGS). Left panels: The increase in *R*^2^ by adding the PGS to the model is shown for PGS calculated at 8 selected *p* value thresholds. The observed p-value is depicted on top of each bar. Right panels: Change in phenotype by PGS quintile is depicted with the lowest quintile as reference. *N* = 140 for all panels

Fast spindle density was positively associated with the SCZ-PGS with the strongest association (incremental *R*^2^ = 5.2%, *p* = 0.0043) for a PT of 0.3 (Fig. 1A, Supplementary Table S1). Fast spindle duration also was positively associated with the SCZ-PGS (strongest association: PT = 0.3; incremental *R*^2^ = 2.9%, *p* = 0.026; Supplementary Table S2) but fast spindle amplitude was not (all *p* > 0.676; Supplementary Table S3).

SCZ-PGS was not associated with slow spindle density (all *p* > 0.493), or slow spindle duration (all *p* > 0.135), and showed a nominally significant negative association with slow spindle amplitude at one PT (PT = 0.001, *R*^2^ = 0.0283, *p* = 0.0472; Supplementary Tables S4–6)).

### Association of fast spindle density with polygenic score for intelligence (IQ)

Fast spindle density was positively associated with the PGS for intelligence with the strongest association (incremental *R*^2^ = 3.3%, *p* = 0.0246) for a PT of 0.2 (Fig. 1B, Supplementary Table S7).

### Association of fast spindle density with polygenic score for schizophrenia: distinction between polymorphisms concordant or discordant for schizophrenia and intelligence

The SCZ-IQ-concordant-PGS was positively associated with fast spindle density (strongest association: incremental *R*^2^ = 5.5%, *p* = 0.00344; PT 0.5; Fig. 1C, Supplementary Table S8). The association of SCZ-IQ-discordant-PGS with fast spindles did not reach significance (strongest association: incremental *R*^2^ = 2.4%, *p* = 0.0576; PT 0.4; Fig. 1D, Supplementary Table S9).

### Association of fast spindle density with polygenic scores for major depression and bipolar disorder

Both Depression and Bipolar-PGS did not show a significant association with fast spindle density (Strongest association: Depression: *R*^2^ = 0.51%, *p* = 0.377, PT = 1, Supplementary Table S10, Bipolar: *R*^2^ = 2.1%, *p* = 0.0717, PT = 0.5, Supplementary Table S11).

### Association of spindle density with *CACNA1I* genetic variance

The *CACNA1I*-based SCZ-score (generated in analogy to the study of Merikanto [14] for explorative analysis) was neither significantly associated with fast spindle density (all *p* > 0.164), fast spindle amplitude (all *p* > 0.320), nor fast spindle duration (all *p* > 0.381; Supplementary Tables S12–14). Additionally, it was neither associated with slow spindle density (all *p* > 0.834), slow spindle amplitude (all *p* > 0.0891) nor slow spindle duration (all *p* > 0.0551; Supplementary Tables S15–S17).

However, the gene-based analysis using MAGMA revealed a nominal significant association between genetic variation in *CACNA1I* and fast spindle density (*p* = 0.0193), but not slow spindle density (*p* = 0.183; Supplementary Table S18). Four SNPs showed association with fast spindle density reaching nominal significance. Information on the included SNPs and their associations with slow and fast spindle density can be found in Supplementary Table S19 and Supplementary Table S20, respectively.

## Discussion

To elucidate the genetic background of impaired spindle activity in schizophrenia, we investigated the association of fast sleep spindle density with the SCZ-PGS in healthy adult subjects. In view of the important role of spindle activity for intelligence and cognition [17–22] we further analyzed the effect of stratification of genetic variance for SNPs concordant and discordant for intelligence and schizophrenia-related risk on this association. We used a genome-wide SCZ-PGS derived from Ripke et al. [2] and a PGS based on a large GWAS for intelligence [49]. In further explorative analyses, we investigated whether these findings were specific to fast versus slow spindles and to schizophrenia compared to bipolar disorder and depression.

We found (1) a positive correlation of PGS for SCZ with fast spindle density and (2) that this association was predominantly driven by the subset of SNPs concordant for schizophrenia and intelligence. The positive association of fast spindle density with genetic background of the disease was specific to schizophrenia: There was no association with PGS for major depression [59] and only a trend level association with PGS for bipolar disorder [58]. Also, association of SCZ-PGS with spindle density was specific to fast but not slow spindle density.

Our finding of a positive correlation of SCZ-PGS with fast spindle density is in line with recent results by Merikanto et al. [14]. In contrast, with regard to the report by Merikanto et al. [14] on an association of slow spindle parameters with a genetic risk score specific for the *CACNA1I* gene, a schizophrenia risk gene coding for the voltage-gated calcium channel subunit alpha 1 known to be implicated in spindle generation [50–52], in a sample of 150 healthy adolescents [14], we did not replicate this result in our explorative analysis in 150 adults. However, we did find preliminary evidence for an association of genetic variance within the *CACNA1I* region with fast spindle density on a gene level. This will have to be further investigated in larger samples.

Our data of a correlation of SCZ-PGS with fast spindle density strengthen the evidence for a genetic background of spindle abnormalities in schizophrenia. They further indicate that the strongest effects of genetic SCZ risk can be observed in fast spindles. Correspondingly, previous data in schizophrenia investigating both spindle types found a fast but not slow spindle deficit [15].

Importantly, both our findings and the findings of the Merikanto study are unexpected with respect to the direction of the correlation of genetic risk for schizophrenia with spindle density as data on spindle abnormalities in patients with schizophrenia consistently report on spindle deficits instead of an increase [15, 16, 32, 33, 40–46, 61]. Findings of opposing direction in healthy subjects compared to patient samples cannot be attributed to the effect of chronic disease course or medication in the patients, as deficient spindle activity is also reported in healthy first-degree relatives of patients with schizophrenia [15, 16, 62] and in untreated first-episode patients [16, 44]. This challenges the current straightforward concept of a genetically mediated spindle deficit in schizophrenia.

**Fig. 2:**
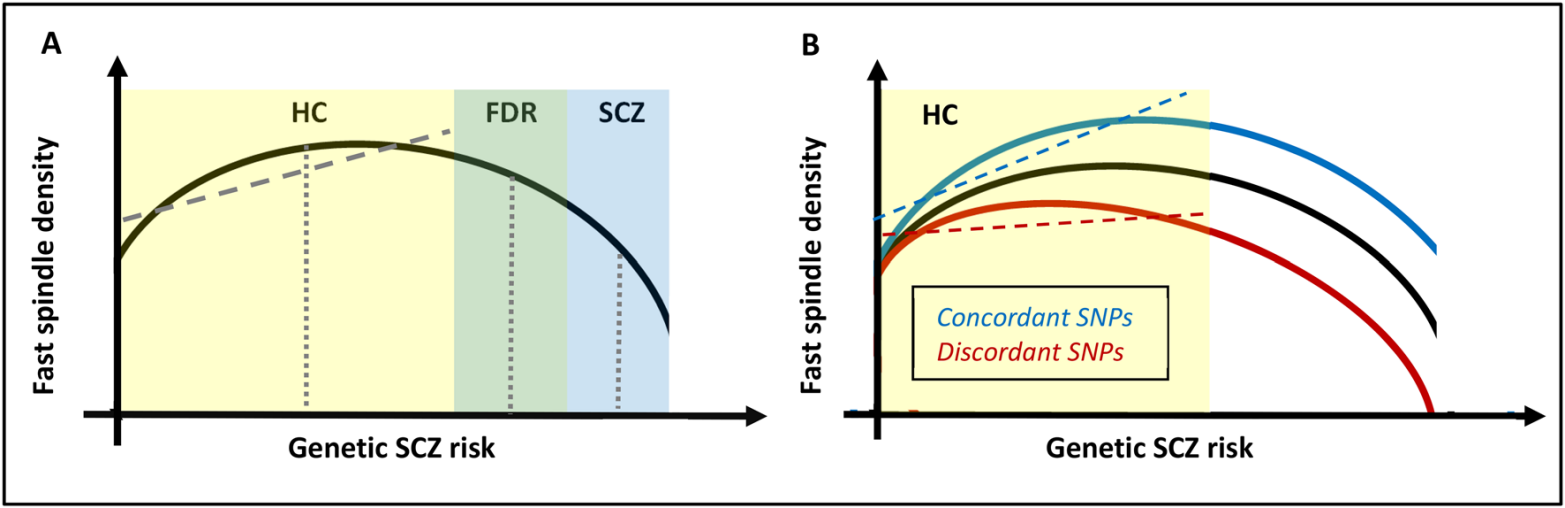
Schematic model of the postulated relationship between fast spindle density and polygenetic risk for schizophrenia (working hypothesis). Yellow areas: refer to data from this study. **A** Model that reconciles the data on fast sleep spindle density in healthy control samples (HC), healthy first-degree relatives (FDR) and patients with schizophrenia (SCZ); dashed line: positive correlation between schizophrenia-PGS and fast spindle density in healthy subjects (first part of this study and [14]); dotted lines: spindle deficit in patient samples and FDR compared to HC (reflecting literature data [15, 46]). **B** Model visualizing the postulated relationship between fast spindle density and SCZ-PGS separately for pro-cognitive variants (SNPs that are concordant for SCZ risk and intelligence; blue curve and blue dashed line) and contra-cognitive variants (SNPs that are discordant for SCZ risk and intelligence; red curve and red dashed line): The positive correlation between spindle density and SCZ-PGS mainly depends on pro-cognitive variants

In view of the recent genetic evidence for a strong role of cognitive function (with educational attainment as a proxy) for identifying genetic heterogeneity within schizophrenia [10] and the important role of spindle activity for cognition [17–22], we analyzed the relation of the correlation between fast spindle density and schizophrenia-PGS to genome-wide genetic variability associated with intelligence (IQ-PGS) [49]. As expected fast spindle density in healthy subjects correlated with the IQ-PGS (which also is a novel finding). Distinction of two subsets of SNPs depending on whether they show concordance or discordance between intelligence and schizophrenia revealed that the positive association of SCZ-PGS with fast sleep spindle density in healthy subjects is mainly driven by polymorphisms concordant for schizophrenia and intelligence. We hypothesize that in healthy subjects the positive association between fast spindle density and SCZ-PGS mainly represents a pro-cognitive part within the spectrum of genetic risk for schizophrenia. As according to Bansal et al. [10] the genetic background of better cognitive function maps to a prognostically more favorable subtype of schizophrenia [10] and the association between high fast spindle density and SCZ-PGS in our study mainly depends on concordant cognitive SNPs (Fig. 1c, see also model in Fig. 2b), intact fast spindle density may be related to a subtype of schizophrenia characterized by genetically determined better cognitive functioning and prognosis. Richard et al. recently showed that cognition in schizophrenia cases was more strongly associated with PGS that index cognitive traits in the general population than with PGS for neuropsychiatric disorders [8]. In line with this, fast spindle density being associated with favourable cognitive traits in the general population [17–22] may be associated with good cognitive function within the clinical spectrum of schizophrenia. This should be further investigated in deeply phenotyped clinical samples of patients with schizophrenia.

As patients with schizophrenia as well as healthy FDR (both having an increased genetic load of schizophrenia-related risk alleles compared to healthy subject samples) show reduced fast spindle density, it could be speculated that the fast spindle density phenotype may follow a converted U-shaped curve with increasing number of schizophrenia risk alleles (Fig. 2a). With regard to an evolutionary framework for a deleterious disorder such as schizophrenia, it previously has been proposed that in healthy subjects inheritance of risk alleles insufficient in number to manifest as schizophrenia may manifest as a behavioral phenotype with adaptive advantages [63] (here high spindle density, high cognitive functioning). A similar relationship has been found for creativity and schizophrenia sharing genetic roots [64]. Possibly, deficient fast spindle density found in patient samples and FDR may be a correlate of a critical number and/or set of risk alleles including their interactions predisposing to actual illness. For direct evidence supporting this association, spindle density and SCZ-PGS will have to be investigated in samples of FDR and unmedicated patients, necessitating larger sample sizes than those of previous investigations of spindle activity in SCZ and FDR [46]. Also, neurobiological pathways mediating the decline of fast spindle density with increasing genetic load for schizophrenia will be interesting to investigate in view of a better understanding of disease mechanisms.

### Strengths and limitations

The good characterization of our sample with exclusion of any sleep or general pathology that could have an influence on the spindle phenotype as well as the study under two-night sleep laboratory conditions are strengths of this work. Although the sample size is rather small for genetic studies, it is the same as in the study by Merikanto and colleagues on this topic [14]. We consider it an indication for the robustness of the present results, that we replicate the main finding of the study by Merikanto et al. [14], the positive association of SCZ-PGS with fast spindle density. Furthermore, the well-defined phenotype of spindle activity with its very strong genetic background allows meaningful results despite the limited sample size. The heterogeneity of algorithms used in spindle research is a potential limitation to the comparability of results. The algorithm used in our study operates with individual spindle peak identification (in contrast to fixed-frequency methods) and, thus, is advantageous for detection of fast and slow spindles taking individual differences in spindle peak-frequency into account [65]. However, future studies should aim to replicate and extend the present results.

## Conclusions

Our results strengthen the evidence for a genetic background of spindle abnormalities in schizophrenia, and fit the considerations about genetically driven cognitive heterogeneity of schizophrenia [8, 10]. In this context, integrity of spindle activity might represent an easily accessible biological marker for a favorable cognitive outcome in schizophrenia. This should stimulate further research on the prognostic value of sleep spindle parameters for clinical subtypes of schizophrenia. Also our results reconcile the previous contradictory findings with regard to the direction of the association of fast spindle density with genetic background for schizophrenia (Fig. 2A, B): As now shown in two studies in independent samples (Merikanto et al. [14] and our present data), healthy subjects with lower polygenic risk for SCZ compared to patient samples and FDR, show a positive correlation between fast spindle density and polygenic risk for SCZ (Fig. 2A), whereby higher fast spindle density mainly maps to a pro-cognitive subset of genetic risk variants (Fig. 2B). Increasing genetic risk for schizophrenia in samples of FDR and patients may then potentially be associated with decreasing fast spindle density resulting in the spindle deficit repeatedly found in clinical samples [32, 33, 40–46] and FDR [15, 16] (Fig. 2A). Within this decline in fast spindle density from healthy subjects to clinical samples, those patients with a genetic background enriched for SNPs concordant for intelligence and SCZ related risk will be expected to show higher spindle density compared to those patients with a genetic background enriched for discordant SNPs (see working hypothesis model in Fig. 2B). This as well as the relation of spindle density with cognitive functioning and prognosis should be further investigated in clinically defined and deeply phenotyped patient samples.

## Supplementary Information

Below is the link to the electronic supplementary material.

Supplementary file1 (DOCX 49 KB)
