# Supplementary material for: Association of polygenic risk for schizophrenia with fast sleep spindle density depends on pro-cognitive variants
Source: Eur Arch Psychiatry Clin Neurosci. 2022 Jun 20;272(7):1193–203. doi: 10.1007/s00406-022-01435-3 (PMC9508216; doi:10.1007/s00406-022-01435-3)
Supplement: Supplementary file 1 — Supplementary file1 (DOCX 49 KB) [file 406_2022_1435_MOESM1_ESM.docx]

**Supplementary Information**

to the Manuscript « **Association of polygenic risk for schizophrenia with fast sleep spindle density depends on pro-cognitive variants** »

submitted to *European Archives of Psychiatry and Clinical Neuroscience*

**Author names:** Claudia Schilling, Lea Zillich, Michael Schredl, Josef Frank, Emanuel Schwarz, Michael Deuschle, Andreas Meyer-Lindenberg, Marcella Rietschel, Stephanie H. Witt, Fabian Streit

**Corresponding author:** Claudia Schilling, MD, Central Institute of Mental Health, Department of Psychiatry and Psychotherapy, Sleep laboratory, Medical Faculty Mannheim / Heidelberg University, J5, D-68159 Mannheim, Germany, Tel. 0049-621-1703-1781; Fax: 0049-621-1703-1785, E-mail: claudia.schilling@zi-mannheim.de, ORCID-No.: 0000-0001-7631-1452

**Table S1:** Schizophrenia-PGS predicting fast spindle density

| **Set** | **Threshold** | **R^2^** | **P** | **Coefficient** | **Standard error** | **Number SNP** |
| --- | --- | --- | --- | --- | --- | --- |
| Base | 0.001 | 0.0135122 | 0.151129 | 91.9661 | 63.7128 | 1786 |
| Base | 0.05 | 0.0317717 | 0.0267642 | 628.944 | 280.962 | 14304 |
| Base | 0.1 | 0.0346614 | 0.0205712 | 907.542 | 387.447 | 21849 |
| Base | 0.2 | 0.0297338 | 0.0322548 | 1115.48 | 515.755 | 33364 |
| Base | 0.3 | 0.0519904 | 0.00432799 | 1779.57 | 613.539 | 42599 |
| Base | 0.4 | 0.0475349 | 0.00644986 | 1964.36 | 710.303 | 50391 |
| Base | 0.5 | 0.0443745 | 0.00856369 | 2117.04 | 793.897 | 57031 |
| Base | 1 | 0.0415996 | 0.0109895 | 2853.89 | 1107.29 | 79507 |

**Table S2:** Schizophrenia-PGS predicting fast spindle amplitude

| **Set** | **Threshold** | **R^2^** | **P** | **Coefficient** | **Standard error** | **Number SNP** |
| --- | --- | --- | --- | --- | --- | --- |
| Base | 0.001 | 0.00107743 | 0.676322 | -498.212 | 1190.86 | 1786 |
| Base | 0.05 | 0.000168676 | 0.868838 | -879.174 | 5314.24 | 14304 |
| Base | 0.1 | 0.00014439 | 0.878569 | -1123.75 | 7341.76 | 21849 |
| Base | 0.2 | 0.000222535 | 0.849561 | -1851.36 | 9742.48 | 33364 |
| Base | 0.3 | 0.000420681 | 0.794239 | 3071.04 | 11752.5 | 42599 |
| Base | 0.4 | 0.000434961 | 0.790854 | 3604.92 | 13567.2 | 50391 |
| Base | 0.5 | 0.000438001 | 0.790141 | 4035.11 | 15133.4 | 57031 |
| Base | 1 | 0.000665988 | 0.742771 | 6927.57 | 21067 | 79507 |

**Table S3:** Schizophrenia-PGS predicting fast spindle duration

| **Set** | **Threshold** | **R^2^** | **P** | **Coefficient** | **Standard error** | **Number SNP** |
| --- | --- | --- | --- | --- | --- | --- |
| Base | 0.001 | 0.00784857 | 0.251175 | 9.96105 | 8.64474 | 1786 |
| Base | 0.05 | 0.0266048 | 0.0333783 | 81.7932 | 38.0664 | 14304 |
| Base | 0.1 | 0.0274658 | 0.0305906 | 114.812 | 52.5576 | 21849 |
| Base | 0.2 | 0.0158818 | 0.101591 | 115.859 | 70.3021 | 33364 |
| Base | 0.3 | 0.0289076 | 0.0264465 | 188.584 | 84.0646 | 42599 |
| Base | 0.4 | 0.0259674 | 0.0356094 | 206.336 | 97.2424 | 50391 |
| Base | 0.5 | 0.0275165 | 0.0304343 | 236.921 | 108.352 | 57031 |
| Base | 1 | 0.0265103 | 0.0336995 | 323.777 | 150.963 | 79507 |

**Table S4:** Schizophrenia-PGS predicting slow spindle density

| **Set** | **Threshold** | **R^2^** | **P** | **Coefficient** | **Standard error** | **Number SNP** |
| --- | --- | --- | --- | --- | --- | --- |
| Base | 0.001 | 0.000585487 | 0.777686 | -20.0493 | 70.8672 | 1786 |
| Base | 0.05 | 0.0015736 | 0.643356 | -146.125 | 314.872 | 14304 |
| Base | 0.1 | 0.00344778 | 0.49286 | -299.963 | 436.194 | 21849 |
| Base | 0.2 | 0.00111579 | 0.696664 | -226.867 | 580.698 | 33364 |
| Base | 0.3 | 0.00170561 | 0.629761 | -338.716 | 700.998 | 42599 |
| Base | 0.4 | 0.00250203 | 0.559213 | -476.368 | 813.611 | 50391 |
| Base | 0.5 | 0.0029203 | 0.528024 | -571.798 | 903.741 | 57031 |
| Base | 1 | 0.00190446 | 0.610457 | -642.088 | 1257.42 | 79507 |

**Table S5:** Schizophrenia-PGS predicting slow spindle amplitude

| **Set** | **Threshold** | **R^2^** | **P** | **Coefficient** | **Standard error** | **Number SNP** |
| --- | --- | --- | --- | --- | --- | --- |
| Base | 0.001 | 0.0282652 | 0.0471885 | -2472.32 | 1234.1 | 1786 |
| Base | 0.05 | 0.0139589 | 0.165094 | -7724.01 | 5533.43 | 14304 |
| Base | 0.1 | 0.0184966 | 0.109535 | -12330.5 | 7653.23 | 21849 |
| Base | 0.2 | 0.0159411 | 0.137706 | -15218.7 | 10190.3 | 33364 |
| Base | 0.3 | 0.0157659 | 0.13991 | -18276.5 | 12306.8 | 42599 |
| Base | 0.4 | 0.0167385 | 0.128145 | -21867.1 | 14282.2 | 50391 |
| Base | 0.5 | 0.015376 | 0.144958 | -23285.6 | 15881.1 | 57031 |
| Base | 1 | 0.0136716 | 0.169549 | -30532.1 | 22105.4 | 79507 |

**Table S6:** Schizophrenia-PGS predicting slow spindle duration

| **Set** | **Threshold** | **R^2^** | **P** | **Coefficient** | **Standard error** | **Number SNP** |
| --- | --- | --- | --- | --- | --- | --- |
| Base | 0.001 | 0.00228898 | 0.550325 | -5.10933 | 8.53246 | 1786 |
| Base | 0.05 | 0.00502394 | 0.375753 | -33.6513 | 37.8636 | 14304 |
| Base | 0.1 | 0.00857372 | 0.246611 | -60.9654 | 52.3855 | 21849 |
| Base | 0.2 | 0.0132802 | 0.1486 | -100.875 | 69.4257 | 33364 |
| Base | 0.3 | 0.0135985 | 0.143775 | -123.266 | 83.819 | 42599 |
| Base | 0.4 | 0.014231 | 0.134695 | -146.425 | 97.2877 | 50391 |
| Base | 0.5 | 0.0119613 | 0.170618 | -149.148 | 108.257 | 57031 |
| Base | 1 | 0.0107244 | 0.194683 | -196.38 | 150.659 | 79507 |

**Table S7:** Intelligence-PGS predicting fast spindle density

| **Set** | **Threshold** | **R^2^** | **P** | **Coefficient** | **Standard error** | **Number SNP** |
| --- | --- | --- | --- | --- | --- | --- |
| Base | 0.001 | 0.032348 | 0.0253927 | 727.394 | 321.918 | 2223 |
| Base | 0.05 | 0.0221249 | 0.0654328 | 1832.79 | 987.041 | 14815 |
| Base | 0.1 | 0.022336 | 0.0641426 | 2554.04 | 1368.77 | 22101 |
| Base | 0.2 | 0.0327168 | 0.0245527 | 4150.64 | 1826.11 | 33223 |
| Base | 0.3 | 0.0303962 | 0.0303536 | 4968.54 | 2271.16 | 42365 |
| Base | 0.4 | 0.0270171 | 0.0414315 | 5364.03 | 2606.25 | 49942 |
| Base | 0.5 | 0.0260183 | 0.0454508 | 5873.38 | 2909.8 | 56733 |
| Base | 1 | 0.0261007 | 0.0451044 | 8058.79 | 3985.99 | 80640 |

**Table S8:** Schizophrenia-Intelligence-concordant-PGS predicting fast spindle density

| **Set** | **Threshold** | **R^2^** | **P** | **Coefficient** | **Standard error** | **Number SNP** |
| --- | --- | --- | --- | --- | --- | --- |
| Base | 0.001 | 0.025688 | 0.0468674 | 99.7833 | 49.7618 | 862 |
| Base | 0.05 | 0.0511624 | 0.00466087 | 643.722 | 223.843 | 7498 |
| Base | 0.1 | 0.040368 | 0.0122784 | 825.116 | 325.239 | 11830 |
| Base | 0.2 | 0.040469 | 0.0121672 | 1128.88 | 444.392 | 18617 |
| Base | 0.3 | 0.0465143 | 0.00706776 | 1465.09 | 535.897 | 24370 |
| Base | 0.4 | 0.0472361 | 0.00662492 | 1743.71 | 632.63 | 29516 |
| Base | 0.5 | 0.054552 | 0.00344157 | 2159.08 | 725.499 | 34131 |
| Base | 1 | 0.0504917 | 0.00494924 | 3169.62 | 1109.95 | 52185 |

**Table S9:** Schizophrenia-Intelligence-discordant-PGS predicting fast spindle density

| **Set** | **Threshold** | **R^2^** | **P** | **Coefficient** | **Standard error** | **Number SNP** |
| --- | --- | --- | --- | --- | --- | --- |
| Base | 0.001 | 0.00150476 | 0.633259 | 25.9926 | 54.3558 | 1155 |
| Base | 0.05 | 0.0045948 | 0.403924 | 194.336 | 232.134 | 8869 |
| Base | 0.1 | 0.0114672 | 0.186314 | 419.48 | 315.855 | 13544 |
| Base | 0.2 | 0.00661325 | 0.31635 | 425.355 | 422.993 | 20804 |
| Base | 0.3 | 0.0224327 | 0.0635604 | 948.052 | 506.957 | 26812 |
| Base | 0.4 | 0.0235069 | 0.0574535 | 1114.32 | 581.704 | 31924 |
| Base | 0.5 | 0.0170305 | 0.106624 | 1047.85 | 645.225 | 36476 |
| Base | 1 | 0.0173971 | 0.102884 | 1569.23 | 955.82 | 54568 |

**Table S10:** Depression-PGS predicting fast spindle density

| **Set** | **Threshold** | **R^2^** | **P** | **Coefficient** | **Standard error** | **Number SNP** |
| --- | --- | --- | --- | --- | --- | --- |
| Base | 0.001 | 0.000133766 | 0.886924 | 23.4436 | 164.566 | 1579 |
| Base | 0.05 | 0.00393382 | 0.440054 | 654.787 | 845.641 | 20044 |
| Base | 0.1 | 0.00108594 | 0.685269 | 500.815 | 1233.15 | 33203 |
| Base | 0.2 | 0.00256077 | 0.533505 | 1096.96 | 1757.35 | 54664 |
| Base | 0.3 | 0.00250374 | 0.538105 | 1346.66 | 2181.88 | 72734 |
| Base | 0.4 | 0.0041646 | 0.426912 | 2105.2 | 2642.04 | 88670 |
| Base | 0.5 | 0.00401771 | 0.435207 | 2414.02 | 3084.77 | 102792 |
| Base | 1 | 0.00514328 | 0.377125 | 4040.67 | 4560.46 | 153787 |

**Table S11:** Bipolar Disorder-PGS predicting fast spindle density

| **Set** | **Threshold** | **R^2^** | **P** | **Coefficient** | **Standard error** | **Number SNP** |
| --- | --- | --- | --- | --- | --- | --- |
| Base | 0.001 | 0.00553743 | 0.359382 | -44.6288 | 48.5326 | 807 |
| Base | 0.05 | 0.00335312 | 0.47603 | 164.007 | 229.5 | 11025 |
| Base | 0.1 | 0.00495615 | 0.385977 | 282.65 | 325.014 | 17963 |
| Base | 0.2 | 0.0145822 | 0.13574 | 727.542 | 484.871 | 29250 |
| Base | 0.3 | 0.0181132 | 0.0959804 | 987.846 | 589.426 | 38375 |
| Base | 0.4 | 0.0205354 | 0.0760756 | 1208.06 | 675.969 | 46133 |
| Base | 0.5 | 0.0211527 | 0.0717402 | 1384.82 | 763.193 | 52993 |
| Base | 1 | 0.0183177 | 0.0941014 | 1802.91 | 1069.6 | 76038 |

**Table S12:** *CACNA1I* based schizophrenia-score predicting fast spindle density

| **Set** | **Threshold** | **R^2^** | **P** | **Coefficient** | **Standard error** | **Number SNP** |
| --- | --- | --- | --- | --- | --- | --- |
| Base | 0.001 | 0.00271528 | 0.521388 | -0.833273 | 1.29626 | 1 |
| Base | 0.05 | 0.0104928 | 0.206319 | 6.0768 | 4.78623 | 5 |
| Base | 0.2 | 0.011366 | 0.188284 | 7.2455 | 5.48021 | 6 |
| Base | 0.3 | 0.0126833 | 0.164396 | 8.96115 | 6.41108 | 7 |
| Base | 1 | 0.0127274 | 0.163658 | 10.2624 | 7.32907 | 8 |

**Table S13:** *CACNA1I* based schizophrenia-score predicting fast spindle amplitude

| **Set** | **Threshold** | **R^2^** | **P** | **Coefficient** | **Standard error** | **Number SNP** |
| --- | --- | --- | --- | --- | --- | --- |
| Base | 0.001 | 0.000200689 | 0.857054 | -4.34608 | 24.0835 | 1 |
| Base | 0.05 | 0.00446659 | 0.394736 | 76.063 | 89.0992 | 5 |
| Base | 0.2 | 0.00553562 | 0.343226 | 97.0071 | 102.002 | 6 |
| Base | 0.3 | 0.00608103 | 0.320419 | 119.04 | 119.382 | 7 |
| Base | 1 | 0.00611157 | 0.319201 | 136.43 | 136.477 | 8 |

**Table S14:** *CACNA1I* based schizophrenia-score predicting fast spindle duration

| **Set** | **Threshold** | **R^2^** | **P** | **Coefficient** | **Standard error** | **Number SNP** |
| --- | --- | --- | --- | --- | --- | --- |
| Base | 0.001 | 0.00250298 | 0.517797 | -0.113698 | 0.175356 | 1 |
| Base | 0.05 | 0.00184935 | 0.578355 | 0.362564 | 0.650818 | 5 |
| Base | 0.2 | 0.00295801 | 0.481934 | 0.525304 | 0.74503 | 6 |
| Base | 0.3 | 0.00453504 | 0.383644 | 0.761526 | 0.871365 | 7 |
| Base | 1 | 0.00458647 | 0.380952 | 0.875513 | 0.996126 | 8 |

**Table S15:** *CACNA1I* based Schizophrenia-score predicting slow spindle density

| **Set** | **Threshold** | **R^2^** | **P** | **Coefficient** | **Standard error** | **Number SNP** |
| --- | --- | --- | --- | --- | --- | --- |
| Base | 0.001 | 0.000323193 | 0.833863 | -0.313561 | 1.49198 | 1 |
| Base | 0.05 | 3.08E-05 | 0.94834 | 0.34986 | 5.38952 | 5 |
| Base | 0.2 | 6.98E-06 | 0.975403 | 0.190935 | 6.18098 | 6 |
| Base | 0.3 | 1.36E-05 | 0.965684 | -0.312229 | 7.24359 | 7 |
| Base | 1 | 1.35E-05 | 0.965809 | -0.355656 | 8.28132 | 8 |

**Table S16:** *CACNA1I* based Schizophrenia-score predicting slow spindle amplitude

| **Set** | **Threshold** | **R^2^** | **P** | **Coefficient** | **Standard error** | **Number SNP** |
| --- | --- | --- | --- | --- | --- | --- |
| Base | 0.001 | 0.0208426 | 0.0891215 | -44.6895 | 26.0935 | 1 |
| Base | 0.05 | 2.21E-03 | 0.581808 | -52.6068 | 95.2837 | 5 |
| Base | 0.2 | 3.35E-03 | 0.497944 | -74.2145 | 109.202 | 6 |
| Base | 0.3 | 3.22E-03 | 0.50613 | -85.3277 | 127.986 | 7 |
| Base | 1 | 3.20E-03 | 0.507458 | -97.248 | 146.323 | 8 |

**Table S17:** *CACNA1I* based Schizophrenia-score predicting slow spindle duration

| **Set** | **Threshold** | **R^2^** | **P** | **Coefficient** | **Standard error** | **Number SNP** |
| --- | --- | --- | --- | --- | --- | --- |
| Base | 0.001 | 0.0232499 | 0.0550591 | -0.342771 | 0.177091 | 1 |
| Base | 0.05 | 0.00598244 | 0.333595 | -0.627982 | 0.647101 | 5 |
| Base | 0.2 | 0.00656545 | 0.310996 | -0.754469 | 0.74183 | 6 |
| Base | 0.3 | 0.00719526 | 0.288752 | -0.925616 | 0.869 | 7 |
| Base | 1 | 0.00711754 | 0.291385 | -1.05249 | 0.993546 | 8 |

**Table S18:** *CACNA1I* gene-based association tests

| **Spindle_measure** | **NSNPS** | **NPARAM** | **N** | **ZSTAT** | **P** | **RSQ** | **RSQ_ADJ** |
| --- | --- | --- | --- | --- | --- | --- | --- |
| Fast spindle density | 33 | 17 | 140 | 2.0677 | 0.019336 | 0.21394 | 0.10441 |
| Slow spindle density | 33 | 17 | 132 | 0.90387 | 0.18303 | 0.16619 | 0.041849 |

*CACNA1I*: CHR:22; BP: 39946758-40105740

**Table S19:** *CACNA1I* single SNP association fast density

| **CHR** | **SNP** | **BP** | **A1** | **TEST** | **NMISS** | **BETA** | **STAT** | **P** |
| --- | --- | --- | --- | --- | --- | --- | --- | --- |
| 22 | rs5757721 | 39947574 | T | ADD | 139 | -0.04 | -0.8231 | 0.412 |
| 22 | rs2413599 | 39952098 | A | ADD | 139 | 0.09057 | 1.849 | 0.0668 |
| 22 | rs3788556 | 39972162 | T | ADD | 139 | -0.01592 | -0.349 | 0.7277 |
| 22 | rs132572 | 39978039 | T | ADD | 139 | -0.249 | -2.999 | 0.003271 |
| 22 | rs132575 | 39982721 | A | ADD | 139 | -0.06705 | -1.417 | 0.1589 |
| 22 | rs73169364 | 39982756 | A | ADD | 139 | -0.1007 | -0.7047 | 0.4823 |
| 22 | rs118028480 | 39988178 | A | ADD | 139 | -0.1 | -0.9176 | 0.3606 |
| 22 | rs2413602 | 39989216 | G | ADD | 139 | 0.008161 | 0.1683 | 0.8666 |
| 22 | rs5750854 | 39990775 | T | ADD | 139 | -0.01826 | -0.3694 | 0.7125 |
| 22 | rs35874709 | 39991692 | T | ADD | 139 | -0.07156 | -0.623 | 0.5344 |
| 22 | rs713767 | 39997710 | G | ADD | 139 | -0.1155 | -0.8406 | 0.4022 |
| 22 | rs5995756 | 40000313 | T | ADD | 139 | -0.02827 | -0.5982 | 0.5508 |
| 22 | rs5757746 | 40000442 | A | ADD | 138 | -0.01629 | -0.3212 | 0.7486 |
| 22 | rs9611204 | 40010383 | A | ADD | 138 | 0.03656 | 0.7909 | 0.4305 |
| 22 | rs3788568 | 40011273 | T | ADD | 139 | 0.02208 | 0.4491 | 0.6541 |
| 22 | rs9607660 | 40016286 | G | ADD | 139 | 0.04938 | 1.077 | 0.2834 |
| 22 | rs136805 | 40018212 | C | ADD | 139 | -0.07595 | -1.45 | 0.1496 |
| 22 | rs111416505 | 40019987 | A | ADD | 139 | -0.03354 | -0.3486 | 0.728 |
| 22 | rs114624981 | 40025446 | G | ADD | 139 | 0.1522 | 0.7067 | 0.4811 |
| 22 | rs117803091 | 40029845 | A | ADD | 139 | -0.2027 | -1.168 | 0.2452 |
| 22 | rs76470176 | 40031771 | T | ADD | 139 | 0.07761 | 0.6038 | 0.5471 |
| 22 | rs136816 | 40033247 | T | ADD | 139 | -0.1136 | -2.043 | 0.04316 |
| 22 | rs136829 | 40046176 | C | ADD | 139 | -0.0731 | -1.344 | 0.1814 |
| 22 | rs136832 | 40046538 | T | ADD | 139 | -0.0731 | -1.344 | 0.1814 |
| 22 | rs8140214 | 40049185 | T | ADD | 139 | -0.2512 | -1.383 | 0.1691 |
| 22 | rs136855 | 40061683 | G | ADD | 139 | -0.07916 | -1.535 | 0.1274 |
| 22 | rs5750870 | 40067640 | G | ADD | 139 | 0.02144 | 0.4659 | 0.6421 |
| 22 | rs5750871 | 40069449 | G | ADD | 139 | 0.1801 | 1.332 | 0.1854 |
| 22 | rs56656729 | 40075733 | A | ADD | 139 | -0.1206 | -0.7281 | 0.4679 |
| 22 | rs58439755 | 40075948 | T | ADD | 139 | 0.28 | 2.45 | 0.01567 |
| 22 | rs3747179 | 40083730 | G | ADD | 138 | -0.0803 | -1.549 | 0.124 |
| 22 | rs5757777 | 40092864 | G | ADD | 139 | 0.00124 | 0.02522 | 0.9799 |
| 22 | rs11704241 | 40102918 | A | ADD | 139 | 0.1347 | 1.981 | 0.04983 |

**Table S20:** *CACNA1I* single SNP association slow density

| **CHR** | **SNP** | **BP** | **A1** | **TEST** | **NMISS** | **BETA** | **STAT** | **P** |
| --- | --- | --- | --- | --- | --- | --- | --- | --- |
| 22 | rs5757721 | 39947574 | T | ADD | 131 | -0.05628 | -1.072 | 0.2859 |
| 22 | rs2413599 | 39952098 | A | ADD | 131 | 0.05885 | 1.107 | 0.2705 |
| 22 | rs3788556 | 39972162 | T | ADD | 131 | -0.04201 | -0.8394 | 0.403 |
| 22 | rs132572 | 39978039 | T | ADD | 131 | 0.09568 | 1.031 | 0.3046 |
| 22 | rs132575 | 39982721 | A | ADD | 131 | -0.02514 | -0.4734 | 0.6368 |
| 22 | rs73169364 | 39982756 | A | ADD | 131 | 0.0353 | 0.2157 | 0.8296 |
| 22 | rs118028480 | 39988178 | A | ADD | 131 | 0.07055 | 0.5649 | 0.5732 |
| 22 | rs2413602 | 39989216 | G | ADD | 131 | -0.03512 | -0.6577 | 0.512 |
| 22 | rs5750854 | 39990775 | T | ADD | 131 | -0.05102 | -0.9351 | 0.3516 |
| 22 | rs35874709 | 39991692 | T | ADD | 131 | -0.0552 | -0.4517 | 0.6523 |
| 22 | rs713767 | 39997710 | G | ADD | 131 | 0.1391 | 0.9556 | 0.3413 |
| 22 | rs5995756 | 40000313 | T | ADD | 131 | 0.004861 | 0.09191 | 0.9269 |
| 22 | rs5757746 | 40000442 | A | ADD | 130 | -0.06195 | -1.109 | 0.2699 |
| 22 | rs9611204 | 40010383 | A | ADD | 130 | 0.01174 | 0.2282 | 0.8199 |
| 22 | rs3788568 | 40011273 | T | ADD | 131 | -0.03799 | -0.7137 | 0.4768 |
| 22 | rs9607660 | 40016286 | G | ADD | 131 | 0.02289 | 0.4488 | 0.6544 |
| 22 | rs136805 | 40018212 | C | ADD | 131 | 0.02662 | 0.4546 | 0.6502 |
| 22 | rs111416505 | 40019987 | A | ADD | 131 | -0.04831 | -0.4665 | 0.6417 |
| 22 | rs114624981 | 40025446 | G | ADD | 131 | -0.2019 | -0.8829 | 0.3791 |
| 22 | rs117803091 | 40029845 | A | ADD | 131 | -0.4072 | -2.228 | 0.02776 |
| 22 | rs76470176 | 40031771 | T | ADD | 131 | 0.003614 | 0.02647 | 0.9789 |
| 22 | rs136816 | 40033247 | T | ADD | 131 | 0.04884 | 0.7939 | 0.4289 |
| 22 | rs136829 | 40046176 | C | ADD | 131 | 0.05507 | 0.9245 | 0.3571 |
| 22 | rs136832 | 40046538 | T | ADD | 131 | 0.05507 | 0.9245 | 0.3571 |
| 22 | rs8140214 | 40049185 | T | ADD | 131 | -0.1853 | -0.8978 | 0.3711 |
| 22 | rs136855 | 40061683 | G | ADD | 131 | 0.03436 | 0.6095 | 0.5434 |
| 22 | rs5750870 | 40067640 | G | ADD | 131 | 0.05704 | 1.13 | 0.2607 |
| 22 | rs5750871 | 40069449 | G | ADD | 131 | -0.03559 | -0.2464 | 0.8058 |
| 22 | rs56656729 | 40075733 | A | ADD | 131 | -0.2748 | -1.422 | 0.1578 |
| 22 | rs58439755 | 40075948 | T | ADD | 131 | 0.2609 | 2.131 | 0.03521 |
| 22 | rs3747179 | 40083730 | G | ADD | 130 | 0.03248 | 0.5737 | 0.5673 |
| 22 | rs5757777 | 40092864 | G | ADD | 131 | 0.02136 | 0.3978 | 0.6915 |
| 22 | rs11704241 | 40102918 | A | ADD | 131 | -0.04118 | -0.554 | 0.5806 |
